# Extracorporeal shockwave therapy in osteoporotic osteoarthritis of the knee in rats: an experiment in animals

**DOI:** 10.1186/ar4601

**Published:** 2014-07-03

**Authors:** Ching-Jen Wang, Chien-Yiu Huang, Shan-Ling Hsu, Jen-Hung Chen, Jai-Hong Cheng

**Affiliations:** 1 Center of Shockwave Medicine and Tissue Engineering, Kaohsiung Chang Gung Memorial Hospital and Chang Gung University College of Medicine, 123 Ta-Pei Road, Niao Sung District, Kaohsiung 833, Taiwan; 2 Department of Orthopedic Surgery, Kaohsiung Chang Gung Memorial Hospital and Chang Gung University College of Medicine, 123 Ta-Pei Road, Niao Sung District Kaohsiung 833, Taiwan

## Abstract

**Introduction:**

This study investigated the effectiveness of extracorporeal shockwave therapy (ESWT) in osteoporotic (OP) osteoarthritis (OA) of rat knee.

**Methods:**

Fifty-six rats were divided into seven groups including sham, OA, OP, OA + OP, OA + ESWT, OP + ESWT, and OA + OP + ESWT groups. The evaluations included gross pathology, bone mineral density (BMD), micro-computed tomography (micro-CT) scan, bone-strength test, histopathologic examination, and immunohistochemical analysis.

**Results:**

On gross pathology, group OA + OP showed larger areas of osteoarthritic changes than did groups OA and OP, as compared with the sham group. BMD and bone strength significantly decreased in groups OA, OP, and OA + OP relative to the sham group, and ESWT significantly improved BMD and bone-strength changes. On micro-CT scan, the subchondral plate thickness significantly decreased, and the bone porosity increased in groups OA, OP, and OA + OP, and ESWT significantly improved the changes in subchondral-plate thickness and bone porosity. In histopathologic examination, Mankin score and safranin O score significantly increased in groups OA and group OA + OP, but not in group OP relative to the sham group, and ESWT significantly improved the changes. In immunohistochemical analysis, Dickkopf-1 (DKK-1) significantly increased, but vessel endothelial growth factor (VEGF), proliferating cell nuclear antigen (PCNA), and bone morphogenetic protein 2 (BMP-2) decreased in groups OA, OP, and OA + OP relative to the sham group, and ESWT significantly reversed the changes.

**Conclusions:**

Osteoporosis increased the severity of cartilage damage in osteoarthritis of the knee. ESWT showed effectiveness in the reduction of osteoporotic osteoarthritis of the knee in rats.

## Introduction

Osteoarthritis (OA) and osteoporosis (OP) are common musculoskeletal disorders. The prevalence of OA and OP escalates with age, especially in women after menopause [1]. OP often manifests in the early stage of OA of the knee, accelerates OA changes with physical inability and functional disability, and directly increases the health-care cost [2-5]. The etiology of OA is multifactorial, including aging, excessive loading to the joint, trauma, infection, overweight, and metabolic disease, and so on [1,6]. OA of the knee is perceived primarily as a cartilage disease accompanied by changes in the subchondral and periarticular bone, such as sclerosis, bone cyst, and osteophyte formation [1,7,8]. Likewise, the etiology of OP is also multifactorial, including estrogen deficiency, disuse atrophy, and infection, and has a direct link to hormone imbalance [9-12]. OP can result in a decrease in bone mineral density, deterioration of bone quality, and microarchitectural fracture of the subchondral bone [13-18]. OP may increase the severity of cartilage damage in OA knee, and the increases in cartilage damage correlate with bone loss and microarchitectural changes [13,14,17,19]. These findings suggest an intimate relation between OP and OA.

Recent studies demonstrated that application of ESWT to subchondral bone of the proximal tibia shows a chondroprotective effect in the initiation of OA of the knee and induces regression of established OA of the knee in rats [20-22]. ESWT was noted to have multifunctional effects in bone and cartilage. DKK-1 promotes *ex vivo* apoptosis of chondrocytes [23]. Our study showed that increased DKK-1 expression correlated with the occurrence of knee OA. Interruption of DKK-1 expression may ameliorate OA-induced chondrocyte apoptosis, cartilage loss, and subchondral bone damage [24], as well as decreased synovitis and hypervascularity in animals with OA knee [25].

In clinical application, ESWT has been effective in the treatment of nonunion of long-bone fractures, and tendinopathy of the shoulder, elbow, knee, and heel. In animal experiments, ESWT was shown to promote bone healing and tissue repair with ingrowth of neovascularization and upregulation of angiogenic and osteogenic growth factors, such as VEGF, endothelial nitric oxide synthase (eNOS), PCNA, BMP-2, and osteocalcin [26]. DKK-1, PCNA, VEGF, and BMP-2 play an important role in wound healing, cartilage repair, and bone synthesis [20,25,27]. For angiogenesis, significant elevations of VEGF, vWF, and FGF basic and a decrease of TGF-β1 were observed. For osteogenesis, BMP-2, osteocalcin, alkaline phosphatase, and IGF were significantly elevated, whereas DKK-1 was decreased. DKK-1 is involved in embryonic development through the inhibition of the Wnt signaling pathway. VEGF is an indication of increased vascular permeability and microvascular activity, including the growth of new vessels. BMP-2 plays an important role in the development of bone and cartilage. These studies demonstrate that ESWT has a potential function of inducing osteoblast differentiation in a variety of cell types. Other studies showed that DKK-1 and Wnt/β-catenin pathways play an important role in the remodeling of subchondral bone, with bone formation and resorption that may link to the development of OP and OA [28,29].

No report has shown an effective method in the prevention or reduction of osteoporotic osteoarthritis of the knee. The purpose of this study was to investigate the effectiveness of ESWT in osteoporotic osteoarthritis of the knee in rats. We hypothesized that ESWT may be effective in the amelioration of osteoporotic osteoarthritis of the knee.

## Methods

### Animal experiment

Fifty-six 8-weeks-old female Sprague–Dawley (SD) rats (BioLASCO, Taipei, Taiwan) were used in the experiment. The experimental protocol of the animal study was approved by the Animal Care Committee of Kaohsiung Chang Gung Memorial Hospital. The animals were kept at the Laboratory Animal Center for 1 week before experiment. The rats were housed at 23°C ± 1°C with a 12-hour light-and-dark cycle and given food and water.

SD rats were randomly divided into seven groups with eight rats in each group. Group 1 was designated the sham group and received sham laparotomy without ovariectomy and sham arthrotomy of the left knee without anterior cruciate ligament transaction (ACLT) or medial meniscectomy (MM). Group 2 was designated the OA group and received ACLT and MM of the left knees [8]. Group 3 was designated the OP group and received bilateral ovariectomies [30]. Group 4 was designated the OA + OP group and received ACLT and MM of the left knee and bilateral ovariectomies. Group 5 was designated the OA + ESWT and received ACLT + MM of the left knee and ESWT. Group 6 was designated the OP + ESWT and received bilateral ovariectomies plus ESWT. Group 7 was designated the OA + OP + ESWT and received ACLT and MM of the left knee, bilateral ovariectomies, and ESWT.

Postoperatively, the animals were cared for by a veterinarian. The surgical site and the activities of the animals were observed daily. After BMD measurement, all animals were killed at 12 weeks after surgery. Left-knee specimens were harvested for examination of gross pathologic lesions, micro-CT scan, bone-strength test, histopathologic examination, and immunohistochemical analysis.

### Anterior cruciate ligament transection and medial meniscectomy

The left knee was prepared in a surgically sterile fashion. Through miniarthrotomy, the ACL was transected with a scalpel, and MM was performed by excising the entire medial meniscus. The knee joint was irrigated, and the incision was closed. Prophylactic antibiotics with ampicillin, 50 mg/kg body weight, were given for 5 days after surgery. Postoperatively, the animals were cared for by a veterinarian. The surgical site and the activities of the animals were observed daily.

### Laparotomy and bilateral ovariectomies

The rats were anesthetized with an intramuscular injection of phenobarbital (50 mg/kg body weight). The abdomen was scrubbed and prepared in surgically sterile fashion. A midline incision was made, and laparotomy was performed. Bilateral ovariectomies were performed by excision of both ovaries. The wound was irrigated and closed in routine fashion.

### Application of shockwave

ESWT was performed in half of animals (eight rats with eight knees in each group) at 1 week after surgery when the wounds healed. The animals were sedated with a 1:1 volume mixture of Rompun (containing xylazine; 5 mg/kg) with Zoletil (containing zolazepam and tiletamine; 20 mg/kg) while receiving ESWT. The source of the shockwave was an OssaTron (Saunwave, Alpharetta, GA, USA). Application of 800 impulses of shockwave at energy-flux density of 0.22 mJ/mm^2^ was given to the proximal medial tibial condyle at 0.5 cm below the joint line and 0.5 cm from the medial skin edge in a single session [27,31,32].

### Bone mineral density

The BMD values within the region of interest (ROI) in medial proximal tibia and distal femur condyle was measured by using dual-energy X-ray absorptiometry (DEXA) (Hologic QDR 4500 W, Hologic, Bedford, MA, USA) before the animals were killed. BMD was used to assess the changes in bone density around the knee in different conditions.

### The score of pathologic lesions

The knee joint was examined under a magnification scope. The gross pathologic lesions with arthritic changes on femoral condoyle and tibial plateau were identified and quantified separately by the semiquantitative scale [19], The severity of joint-surface damage was scored and categorized as follows: (a) normal, (b) discoloration, mild surface irregularities or pitting, (c) partial-thickness erosion or fibrillation, and (d) full-thickness erosion and/or osteophytes. The overall scores were obtained by summing the cartilage scores of the lesions in femoral condoyle and tibial plateau cartilages in eight knees of each group [19].

### Micro-CT scan

The proximal part of the tibia and the distal part of the femur were scanned by a micro-CT scanner (Skyscan 1076; Skyscan, Luxembourg, Belgium) with isotropic voxel size of 36 × 36 × 36 μm^3^, as previously described [33]. In brief, the X-ray voltage was set at 100 kV, and the current, at 100 μA. X-ray projections were obtained at 0.75-degrees angular step with a scanning angular range of 180 degrees. Reconstructions of the image slices were performed with NRecon software (Skyscan), and the process generated a series of planar transverse gray-value images. Volume of interest (VOI) of bone morphometry was selected with a semiautomatic contouring method by Skyscan CT-Analyser Software (Skyscan). Three-dimensional cross-sectional images of the femoral and tibial subchondral bone regions were generated by CTVol v2.0 software. The subchondral porosity was defined as the ratio of the volume of the pores to the total volume of the subchondral region. The subchondral plate thicknesses of the femur and tibia were measured by individual point-to-point distance from the top of the cartilage to the subchondral bone plate by averaging at least six measurements per sample.

### Bone-strength test

The distal femur and proximal tibia bones were harvested for bone-strength tests, including peak load and breaking moment. A 3-cm bone from the medial proximal tibia and distal femur was obtained. The bone specimens were subjected to bone-strength tests on a Material Testing System (MTS, Synergie 200; Canton, MA, USA) machine. The peak compression strength and the breaking moment of inertia were measured by using the slow-load compression technique until fracture occurred.

### Histopathologic examination

The bone and cartilage of the knee were subjected to histopathologic examination. The harvested specimens are fixed in 4% PBS-buffered formaldehyde and decalcified in 10% PBS-buffered EDTA at room temperature for about 2 months. Decalcified specimens were subjected to paraffin-wax embedding and dissection into 5-μm-thick sections. The specimens were stained with hematoxylin-eosin (H&E) stain and safranin O staining. The degenerative changes of the cartilage were graded histologically by using the Mankin scoring system for the assessments of cartilage structure, cartilage cells, and tidemark integrity, with a score from 0 to 15 [7]. In addition, a safranin O score was obtained according to the Osteoarthritis Research Society International (OARSI) cartilage OA grading system [34]. The scores were obtained on a 0–to-24 scale by multiplying the index of grades with stages.

### Immunohistochemical analysis

The harvested knee specimens were fixed in 4% PBS-buffered formaldehyde for 48 hours and decalcified in 10% PBS-buffered EDTA solution. Decalcified tissues were embedded in paraffin wax. The specimens were cut longitudinally into 5-μm-thick sections and transferred to polylysine-coated slides (Thermo Fisher Scientific, Waltham, MA, USA). The immunohistochemical stains were done by following the protocol provided in the immunostaining kit (Abcam, Cambridge, MA, USA). In brief, tissue sections were deparaffinized in xylene, hydrated in graded ethanol, and treated with peroxide-block and protein-block reagents. Sections of the specimens were immunostained with specific antibodies for DKK-1 (Cell Signaling Technology, Danvers, MA, USA), PCNA (Santa Cruz Biotechnology, Santa Cruz, CA, USA), VEGF (Santa Cruz Biotech), and BMP-2 (Abcam) for overnight to identify the angiogenesis and osteogenesis biomarkers according to the product immunohistochemical staining protocols. The immunoreactivity in specimens was demonstrated by using a goat anti-rabbit horseradish peroxidase (HRP)-conjugated and 3′,3′-diaminobenzendine (DAB), which were provided in the kit. The immunoactivities were quantified from five areas in three sections of the same specimen by using a Zeiss Axioskop 2 plus microscope (Carl Zeiss, Gottingen, Germany). All images of each specimen were captured by using a cool CCD camera (Media Cybernetics, Silver Spring, MD, USA). Images were analyzed by using an Image-Pro Plus image-analysis software (Media Cybernetics). The percentages of immunolabeled positive cells over the total cells were calculated from articular cartilage and subchondral bone of distal femur and proximal tibia.

### Statistical analysis

SPSS ver. 17.0 (SPSS Inc., Chicago, IL, USA) was used in statistical analysis. Data were expressed as mean ± SD. One-way ANOVA and Tukey tests were used to compare sham versus OA, OP, and OA + OP with ESWT and without ESWT (designated as _*_P < 0.05 and _**_P < 0.001). Paired sample *t* tests were used to compare ESWT groups and without-ESWT groups (designated as #*P* < 0.05 and ##P < 0.001) and independent-sample *t* tests were used to compare OA versus OA + OP and OA + ESWT versus OA + OP + ESWT (designated as ※*P* < 0.05 and ※※*P* < 0.001). The *P* values of less than 0.05 were considered significant.

## Results

### ESWT reduces arthritic area of injury joint

**Figure 1:**
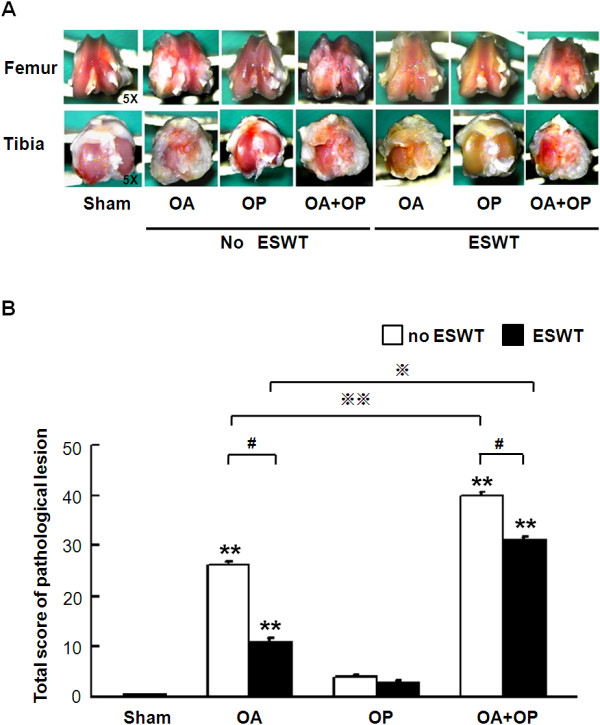
**Gross pathologic lesions in a rat model of OA knee in sham, OA, OP, and OA + OP groups with and without ESWT (A).** The scores of the pathologic lesions were measured and calculated, and the results showed significantly higher lesion scores in OA and OA + OP groups as compared with sham (indicated as _**_P < 0.01). ESWT significantly decreased the total score of pathologic lesions as compared with without ESWT (indicated as #*P* < 0.05). The OA + OP group has significantly higher lesion score than the OA group with and without ESWT, respectively **(B)** (indicated as ※*P* < 0.05 and ※※*P* < 0.001); *n* = 8.

Significant difference in the sizes of gross arthritic lesions of the knee was noted between ESWT and non-ESWT. The gross pathologic lesions with and without ESWT are shown in Figure 1A. With no ESWT, the sham group showed smooth surfaces with no surface irregularity or pits. The OA group demonstrated broad areas of articular surface destruction with fibrous tissue thickening around the tibia. The OP group showed intact surfaces with decreased surface brightness and inflammation. The OA + OP group showed severe articular surface erosion, ulcerations, pitting, and osteophytes with proliferation of fibrous tissue. With ESWT, the OA group showed thickening of the articular surface with yellowish discoloration but no osteophytes. The OP group showed intact surfaces with no ulceration or discoloration. The OA + OP group demonstrated osteoarthritic changes including articular-surface erosions, ulcerations, pitting, and osteophyte formation. The statistical comparisons between ESWT and no ESWT are shown in Figure 1B; the OA and OA + OP groups showed significantly larger areas than the sham group. The OA and OA + OP group with no ESWT showed significantly larger lesions than OA and OA + OP with ESWT. The OA + OP group with ESWT showed significantly larger lesions than did those with no ESWT. It appears that ESWT is effective to ameliorate the severity of osteoporotic osteoarthritis in rat knee.

### ESWT enhances BMD and bone strength

**Figure 2:**
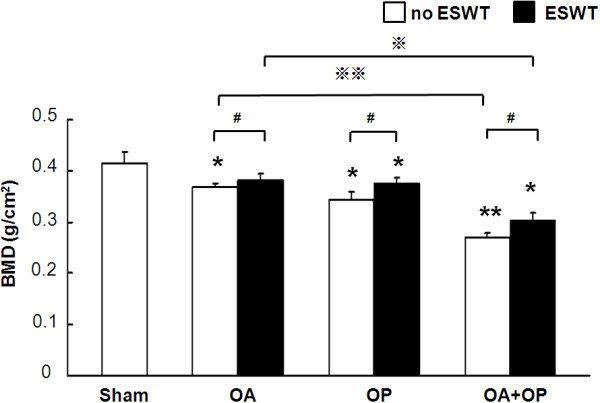
**The BMD values were measured from the region of interest in the knee.** BMD values significantly decreased in OA, OP, and OA + OP groups as compared with sham without ESWT. ESWT significantly increased the BMD values in OA, OP, and OA + OP groups relative to sham (indicated as _*_P < 0.05 and _**_P < 0.001). The BMD values in animals treated with ESWT showed significant improvement as compared with animals without ESWT (indicated as #*P* < 0.05). The OA group showed significantly higher BMD values than the OA + OP group, with and without ESWT, respectively (indicated as ※*P* < 0.05 and ※※*P* < 0.001); *n* = 8.

**Figure 3:**
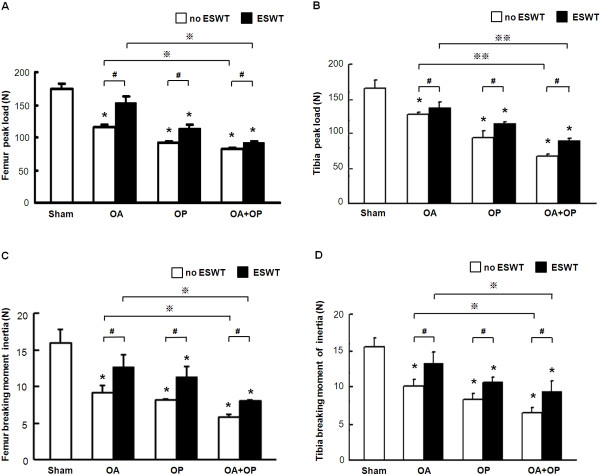
**Bone-strength tests included peak load and breaking moment of inertia with and without ESWT.** The peak load in femur **(A)** and tibia **(B)** showed significantly higher values in the OA group as compared with OP and OA + OP, with and without ESWT, respectively (indicated as _*_P < 0.05, ※*P* < 0.05, and ※※*P* < 0.001). The animals with ESWT showed significantly greater values than did the animals without ESWT (indicated as #*P* < 0.05). The breaking moment of inertia in the femur **(C)** and tibia **(D)** demonstrated significantly higher values in the OA group than in the OP and OA + OP groups, with and without ESWT, respectively (indicated as _*_P < 0.05_,_ ※*P* < 0.05). ESWT-treated animals showed a significantly higher breaking moment than did those without ESWT (indicated as #*P* < 0.05); *n* = 8.

BMD values were measured with and without ESWT (Figure 2). BMD significantly decreased in groups OA, OP, and OA + OP, as compared with sham. ESWT significantly increased BMD in OA, OP, and OA + OP, as compared with sham. BMD was significantly higher with ESWT than without ESWT in OA, OP, and OA + OP groups.The bone-strength tests are shown in Figure 3. The peak load (Figure 3A,B) and the breaking moment of the femur and tibia (Figure 3C,D) significantly decreased in groups OA, OP, and OA + OP relative to sham group, with the most decrease in group OA + OP. ESWT significantly improved the peak load and breaking moment in groups OA, OP, and OA + OP as compared with the sham group. The results showed that ESWT significantly improved the BMD value and bone strength in osteoporotic osteoarthritis of the knee in rats.

### ESWT improves subchondral plate thickness and bone porosity

**Figure 4:**
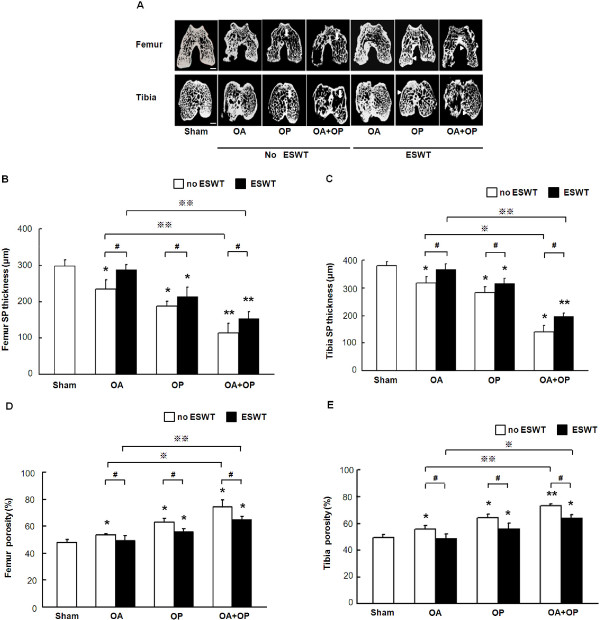
**Micro-CT images showed morphology and distribution of subchondral bone of distal femur and proximal tibia with and without ESWT (A).** The subchondral plate thicknesses of femur **(B)** and tibia **(C)** were significantly reduced in OA, OP, and OA + OP groups as compared with sham (indicated as _*_P < 0.05 and _**_P < 0.001). ESWT-treated groups showed significantly higher values than did those without ESWT (indicated as #*P* < 0.05). The OA group showed significantly thicker subchondral plates than did the OA + OP group, with and with ESWT, respectively (indicated as ※*P* < 0.05 and ※※P < 0.001). The bone porosities of femur **(D)** and tibia **(E)** showed increased bone porosity in OA, OP, and OA + OP groups as compared with sham with or without ESWT (indicated as _*_P < 0.05). ESWT significantly reduced the bone porosity in groups OA, OP, and OA + OP, as compared with sham (indicated as #*P* < 0.05). The OA group showed significantly lower bone porosity than did the OP and OA + OP groups with ESWT and without ESWT, respectively (indicated as ※*P* < 0.05 and ※※*P* < 0.001). The scale bar represents 1 mm; *n* = 8.

The results of micro-CT scan are summarized in Figure 4. The subchondral plate thickness significantly decreased, and bone porosity increased in distal femur and proximal tibia in groups OA, OP, and OA + OP, with the most decrease noticed in group OA + OP as compared with sham. ESWT significantly improved the subchondral plate thickness and bone porosity in groups OA, OP, and OA + OP, especially in group OA + OP. ESWT seems more effective in the improvement of subchondral plate thickness and bone porosity in group OA + OP than in group OA.

### ESWT reduces cartilage damage in knee osteoarthritis

**Figure 5:**
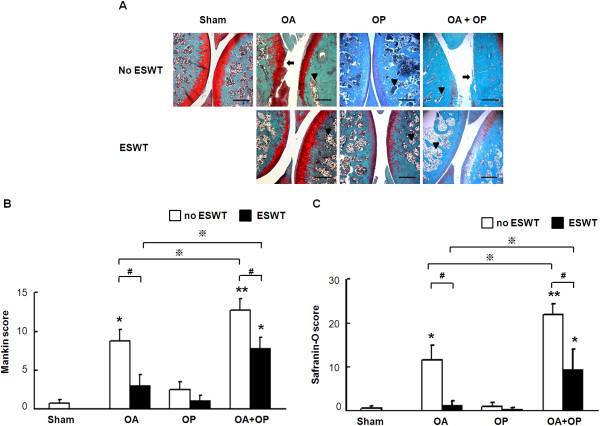
**Histopathologic examination showed the cartilage damages in osteoarthritis of the knee, as shown in arrows with and without ESWT (A).** Mankin score **(B)** and safranin O **(C)** score significantly increased in OA and OA + OP groups, as compared with sham with and without ESWT (indicated as _*_P < 0.05). ESWT significantly reduced Mankin and safranin O scores (indicated as #*P* < 0.05). Mankin and safranin O scores were significantly higher in the OA + OP group than in the OA group. ESWT, significantly decreased Mankin and safranin O scores in OA and OA + OP group (indicated as ※*P* < 0.05). The scale bar represents 200 μm; *n* = 8. Arrow indicates cartilage injury. Arrowhead indicates spongy bone.

The microscopic features of histopathologic examination of groups OA, OP, and OA + OP are shown in Figure 5A. The graphic illustrations with and without ESWT are shown in Figure 5B for Mankin score and Figure 5C for safranin O stain. Significant cartilage damage associated with elevations of Mankin score and safranin O stain were observed in groups OA and OA + OP, but not in group OP, as compared with sham. ESWT significantly improved the cartilage changes with decreases in Mankin score and safranin O stain in groups OA and OA + OP. (Figures 5B,C). These findings suggest that the ESWT shows chondroprotective effect in groups OA, OP, and OA + OP.

### ESWT causes favorable molecular changes in knee osteoarthritis

**Figure 6:**
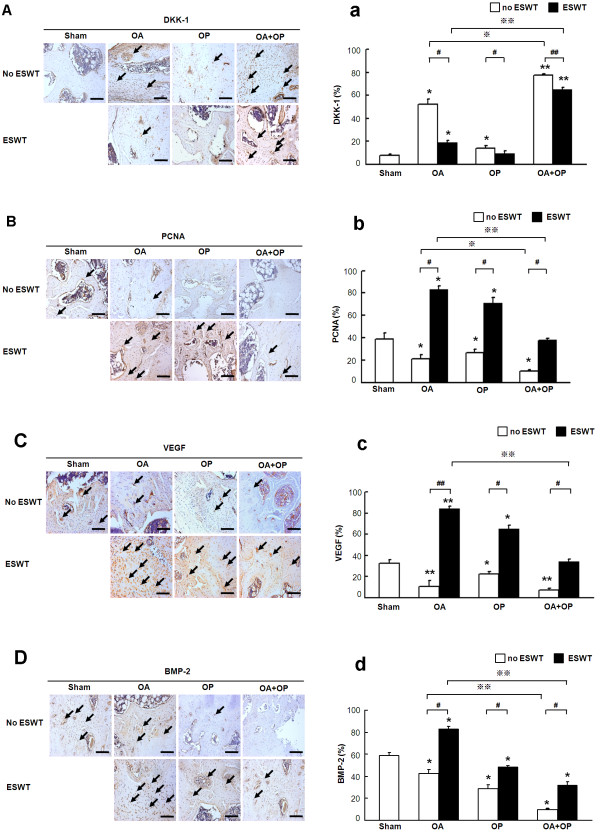
**Immunohistochemical analysis for molecular changes in osteoarthritis of the knee with and without ESWT in representative images of microscopic features, and the results, in graphs.** DKK-1 expressions **(Aanda)** were significantly higher in OA, and OA + OP groups, but not in the OP group as compared with the sham group (indicated as _*_P < 0.05 and _**_P < 0.001). ESWT significantly decreased the DKK-1 expressions, as compared with those without ESWT (indicated as #*P* < 0.05 and ##*P* < 0.001). DKK-1 expression was significantly higher in the OA + OP group than in the OA group and decreased after ESWT (indicated as ※*P* < 0.05 and ※※*P* < 0.001). The expressions of PCNA **(Band b)**, VEGF **(C and c)** and BMP-2 **(D and d)** were significantly decreased in the OA, OP, and OA + OP groups, and the data increased after ESWT (indicated as _*_P < 0.05 and _**_P < 0.001). ESWT significantly increased PCNA, VEGF, and BMP-2 expressions, as compared with those without ESWT (indicated as #*P* < 0.05 and ##*P* < 0.001). The PCNA, VEGF, and BMP-2 expressions in the OA group significantly decreased as compared with the OA + OP group after ESWT (indicated as ※*P* < 0.05 and ※※*P* < 0.001). The scale bar represents 50 μm; *n* = 8. The arrows indicate the level of molecular expression in subchondral bone.

Immunohistochemical analysis of DKK-1, PCNA, VEGF, and BMP-2 in subchondral bone and articular cartilage in OA, OP, and OA + OP with and without ESWT is shown in Figure 6A-D. The representative images of microscopic features are shown in Figure 6a-d. Significant increases in DKK-1 expression (Figure 6A) and decreases in PCNA (Figure 6B), VEGF (Figure 6C), and BMP-2 (Figure 6D) expressions were noted in groups OP and OA + OP with and without ESWT, as compared with the sham group. ESWT significantly improved the changes in DKK-1, PCNA, VEGF, and BMP-2, with the most changes noticed in group OA + OP. These results demonstrated that ESWT modulates the key factors for joint repair in groups OA, OP, and OA + OP.

## Discussion

The most significant findings of the current study revealed that OP increases the severity of cartilage damage in OA of the knee in rats. Osteoporotic OA resulted in the most severe form of arthritic changes of the knee, as shown on gross pathology, BMD, bone strength, micro-CT scan, histopathologic examination, and immunohistochemical analysis. The relation between osteoporosis and osteoarthritis remains debated. Some studies reported that OP increased the severity of cartilage damage in OA [19]. Others reported an inverse relation between bone density and bone turnover in patients with OA and OP [13-18].

OP is often present in an early stage of osteoarthritis with bone loss and microarchitectural damage or trabecular fracture in subchondral bone [15,18]. As the subchondral bone fracture heals, it often results in less compliant and harder bone tissue that in turn causes degenerative changes of the articular cartilage [35-37]. Many studies, including the current study, reported a close relation between OP and OA [13-16].

In this study, ESWT showed beneficial effects in osteoporotic OA in rat knee. Prior studies reported that ESWT has chondroprotective effects in ACLT-induced OA knee in rats [20-22]. Zhao and his colleagues [22] reported that ESWT reduces the progression of knee OA in rabbits by reducing the nitric oxide level and chondrocyte apoptosis. The results of the current study are in agreement with others that shockwave therapy showed chondroprotective effects in the development of OA changes of the knee in animal experiment [20,27,31,32].

Prior studies showed that application of ESWT to the subchondral bone of the medial proximal tibia results in a chondroprotective effect associated with improved subchondral bone remodeling [28,30]. The relation between subchondral bone changes and the initiation and progression of OA changes in the knee remains debated. Emerging evidence showed that subchondral bone changes may play a role in OA changes of the knee and raised the possibility that early intervention to the subchondral bone may reduce or retard the initiation or progression of knee OA [9,10,35-37].

The results of the current study showed that early intervention with ESWT resulted in chondroprotective effect in the initiation of OA changes of the rat knee.

The functional integrity of the articular cartilage relies on the mechanical property of the subchondral bone [35,37]. Radon and his colleagues [35] proposed the potential role of subchondral bone in the initiation and progression of OA knees. Remodeling of subchondral bone plate that is exposed to excessive no physiological mechanical load results in stiffer bone of inhomogeneous density with poor shock absorption. The denser and less compliant bone can generate shear stress that alters the physiological deformation and cartilage damage. It was suggested that increased subchondral bone stiffness can reduce the ability of the knee joint to dissipate the load and distribute the forces within the joint, and subsequently increases the force loads on the overlying articular cartilage, which in turn accelerates the cartilage damage over time [13-17].

Emerging evidence indicates that bone turnover increases in patients with early OA changes of the knee [9,10]. As the OA changes progressed, increased bone resorption and reduced subchondral bone volume occur, and finally, the process is followed by increased bone formation and increased subchondral bone volume (sclerosis) and formation of periarticular osteophytes [18,37]. It appears that a close link exists between OP and OA. The OA of the knee is perceived primarily as a cartilage disease; however, many recent studies, including the current study, raised the possibility that subchondral bone changes may precede the articular cartilage changes in the development of OA of the knee [15,16,37].

Some limitations exist in this study. The results of this study are based on studies in a limited number of small rats. The results may vary in larger animals and human subjects. The radiographic appearance of the knee was not shown because radiographs are insensitive and inaccurate in the quantitative assessment of OA of the knee in small animals. The ESWT dose was based on a pilot study in small animals, and validation of shockwave dosage may be necessary.

## Conclusion

OP increases the severity of cartilage damage in OA of the knee in rats. ESWT is effective in the amelioration of osteoporotic OA of the knee in rats.

## Abbreviations

ACLT: Anterior cruciate ligament transection; BMD: bone mineral density; BMP-2: bone morphogenetic protein 2; DAB: 3′,3′-diaminobenzedine; DEXA: dual-energy x-ray absorptiometry; DKK-1: Dickkopf-1; eNOS: endothelial nitric oxide synthase; ESWT: extracorporeal shockwave therapy; HRP: horseradish peroxidase; micro-CT: micro computed tomography; MM: medial meniscectomy; OA: osteoarthritis; OP: osteoporosis; OVX: ovariectomy; PCNA: proliferating cell nuclear antigen; ROI: region of interest; SD rat: Sprague–Dawley rat; VEGF: vessel endothelial growth factor.

## Competing interests

The authors declared that they did not receive any honoraria or consultancy fee in the writing of this article. No fund was received or will be received from a commercial party related to the subject in this article. One author (CJW) serves as a member of the advisory committee of Sanuwave. Alpharetta GA. The remaining authors declared no conflict of interest.

## Authors’ contributions

C-JW participated in the study with primary duties including the conception and design of the study, data analysis and interpretation of data, drafting the article, and final proof of the revised manuscript. C-YH participated in the study with primary duties in the performance of animal experiment, BMD measurement, bone-strength test, micro-CT scan, histopathologic examination and immunohistochemical analysis, data collection and statistical analysis, and final proof of the revised manuscript. J-HC participated in the study with primary duties in reference search, review, collection, and analysis of data, and final proof of the revised manuscript. S-LH participated in the study with primary duties in data acquisition, analysis, and reference search and final proof of the revised manuscript. J-HC participated in the study with primary duties in the performance of animal experiments, reference search, data analysis and revision of the manuscript, and final proof of the revised manuscript. All authors read and approved the final manuscript.
